# A retrospective study of patient-tailored FOLFIRINOX as a first-line chemotherapy for patients with advanced biliary tract cancer

**DOI:** 10.1186/s12885-020-07004-y

**Published:** 2020-06-03

**Authors:** Ayhan Ulusakarya, Abdoulaye Karaboué, Oriana Ciacio, Gabriella Pittau, Mazen Haydar, Pamela Biondani, Yusuf Gumus, Amale Chebib, Wathek Almohamad, Pasquale F. Innominato

**Affiliations:** 1 Assistance Publique-Hopitaux de Paris, Department of Medical Oncology, Paul Brousse Hospital, 12-14 Avenue Paul Vaillant Couturier, 94800 Villejuif, France; 2 INSERM U935 Campus CNRS, Villejuif, France; 3 Medical oncology unit, GHI Le Raincy-Montfermeil, Montfermeil, France; 4 Assistance Publique-Hôpitaux de Paris, Department of Surgery, Paul Brousse Hospital, Centre Hépato-Biliaire, Villejuif, France; 5 North Wales Cancer Centre, Ysbyty Gwynedd, Betsi Cadwaladr University Health Board, Bangor, UK; 6 Cancer Chronotherapy Team, Cancer Research Centre, Division of Biomedical Sciences, Warwick Medical School, Coventry, UK

**Keywords:** FOLFIRINOX, Advanced biliary tract cancer, Real life practice, Cholangiocarcinoma

## Abstract

**Background:**

FOLFIRINOX is a pillar first-line regimen in the treatment of pancreatic cancer. Historically, biliary tract cancer (BTC) and pancreatic cancer have been treated similarly with gemcitabine alone or combined with a platinum compound. With growing evidence supporting the role of fluoropyrimidines in the treatment of BTC, we aimed at assessing the outcomes of patients (pts) with BTC on frontline FOLFIRINOX.

**Methods:**

We retrospectively analyzed data of all our consecutive patients with locally advanced (LA) or metastatic (M) BTC who were registered to receive FOLFIRINOX as a first-line therapy between 12/2013 and 11/2017 at Paul Brousse university hospital. The main endpoints were Overall Survival (OS), Time-to-Progression (TTP), best Objective Response Rate (ORR), Disease Control rate (DCR), secondary macroscopically-complete resection (res) and incidence of severe (grade 3–4) toxicity (tox).

**Results:**

There were 17 male (40%) and 25 female (60%) pts. aged 36 to 84 years (median: 67). They had PS of 0 (55%) or 1 (45%), and intrahepatic cholangiocarcinoma (CCA) (21 pts., 50%), gallbladder carcinoma (8 pts., 19%), perihilar CCA (7 pts., 17%), distal CCA (4 pts., 10%) and ampulloma (2 pts., 5%). BTC was LA or M in 10 (24%) and 32 pts. (76%) respectively. Biliary stent was placed in 14 pts. (33%). A median of 10 courses was given with median treatment duration of 6 months. There were no untoward toxicity issues, with no febrile neutropenia, emergency admission for toxicity or toxic death. We observed 12 partial responses (29%) and 19 disease stabilisations (45%). Six patients (14%) underwent secondary R0-R1 resection. Median TTP was 8 months [95%CL, 6–10] and median OS was 15 months [13–17]. Patients undergoing secondary resection displayed a 3-y disease-free rate of 83%.

**Conclusions:**

First-line FOLFIRINOX offers promising results in patients with LA and M-BTC. It deserves prospective evaluation to further improve outcomes for advanced BTC.

## Background

Biliary tract cancer (BTC) is a heterogeneous group of cancers which includes intrahepatic cholangiocarcinoma (IHCC), extrahepatic cholangiocarcinoma, gallbladder cancer, and ampulloma. BTC is one of the most lethal neoplastic diseases, with very similar incidence and mortality rates, and it is now the sixth leading cause of cancer-related death worldwide [1]. Only surgery can provide a chance for better long term outcomes, but most of the patients initially present with an advanced and inoperable disease [2, 3]. Potentially curative resection is feasible in about only 20% of patients at initial diagnosis [4]. However, operable patients often have recurrent disease after curative surgery. Indeed, the postoperative median recurrence-free survival is reported to be of the order of 18–24 months even in very recent trials [5, 6]. Therefore, while waiting for more effective targeted therapies for at least some subsets of these patients [7], palliative chemotherapy continues having a crucial role to improve the outcome of patients with advanced BTC.

At present, the standard first-line chemotherapy regimen for advanced BTC is the combination of gemcitabine and cisplatin, which provides a survival advantage as compared with gemcitabine alone [8–10]. However, other regimens are being developed with the aim of broadening the therapeutic index of the gemcitabine-cisplatin combination. Historically, BTC and pancreatic cancers were treated similarly with gemcitabine alone or combined with a platinum compound. Previous studies have shown that the combination of 5-Fluorouracil, leucovorin, oxaliplatin and irinotecan (FOLFIRINOX) or the combination of gemcitabine and nab-paclitaxel are associated with improved survival compared with gemcitabine alone in patients with metastatic pancreatic ductal adenocarcinoma [11–13]. Thus, both regimens are the standard of care of locally advanced or metastatic pancreatic cancer. However, FOLFIRINOX appears as the most active of the two regimens, in terms of overall survival (OS), time to progression (TTP), and objective response (OR). Multiple reports have moreover demonstrated some degree of activity of fluoropyrimidines against BTC, both in the advanced and in the adjuvant setting [14].

Given the fairly limited number of reports on FOLFIRINOX in BTC [15–18], we decided to perform a retrospective analysis of the outcomes of all consecutive patients with advanced BTC treated with FOLFIRINOX as first-line chemotherapy at Paul Brousse university hospital, Villejuif, France over a 4-year period.

## Methods

### Study objectives

The main objective of the study was to retrospectively evaluate the outcomes of routine administration of FOLFIRINOX as first line systemic treatment in patients with advanced BTC.

### Patient selection

Every patient having received at least one cycle of FOLFIRINOX regimen as a first line therapy against unresectable, locally advanced or metastatic BTC, from December 2013 to November 2017 at the department of Oncology at Paul Brousse University Hospital (Villejuif, France) was selected from hospital pharmacy log-out registries. Criteria for inclusion in this real-world data report were therefore limited to a formal diagnosis of advanced BTC and to treatment with FOLFIRINOX. Since no ethical concern was identified in this retrospective evaluation of patients’ outcomes, the study was exempt from ethical review, according to the current legislation in France.

### Study treatment

FOLFIRINOX regimen consisted in the intravenous administration of irinotecan (180 mg/m^2^), oxaliplatin (85 mg/m^2^), leucovorin (400 mg/m^2^) and 5FU (2800 mg/m^2^, including the bolus injection and the 46-h constant infusion) [11]. The dosing was individualized according to patient profile as per our experience in the treatment of advanced pancreatic adenocarcinoma, with a “patient-tailored” approach [13]. Consequently, chemotherapy could have been started with or without irinotecan and/or with dose reductions of the cytotoxics in selected instances. Chemotherapy was started without irinotecan after biliary stenting in case of elevated serum bilirubin. Irinotecan was added when bilirubinemia became ≤1.5 of upper limit of the normal. In case of grade 2 or more sensory neuropathy, oxaliplatin was withhold. Treatment was initiated with decreased doses in patients with clinical characteristics of frailty (e.g. age, comorbidities) [19]. Patient-tailored FOLFIRINOX was discontinued in case of secondary surgery, disease progression or occurrence of an unacceptable toxicity. Recommended supportive care drugs were administered to all patients to prevent or treat chemotherapy-related toxicities as described [13, 20].

Patients underwent fortnightly full biology work-up, including full blood count and renal and hepatic biochemistry, prior to any new chemotherapy administration. Every 3rd to 4th cycle, comparative imaging was performed, including a triple-phase contrasted thoraco-abdomino-pelvic CT scan and if indicated, hepatic MRI and/or 18Fluorodeoxyglucose PET scan. In case of disease control, the indication and feasibility of secondary surgical resection was discussed at the hepatobiliary onco-surgical multidisciplinary team meeting [21].

### Assessments

The chief investigator (AU) collected all the clinical parameters from the patients’ medical records. Baseline demographic and clinical characteristics, actual chemotherapy drug doses administered and number of cycles, as well as outcome measures of antitumor response, TTP, OS, secondary surgical resection and serious adverse events according to NCIC-CTCAE v3.0 [22] were entered in a full anonymous form into the dedicated database for analysis. An independent investigator (AK) conducted a quality-review of the collected data to ensure consistency and plausibility. In case of discrepancies, an agreement was reached after discussion with the responsible physician.

### Statistical consideration

The primary endpoint was the descriptive report of real-world data from all consecutive patients treated with FOLFIRINOX. Efficacy was assessed with RECIST criteria [23] for best OR, macroscopically-complete (R0 or R1) secondary surgical resection rate, as well as with time-to-event outcomes. TTP and OS were computed using Kaplan-Meier method and were defined as the time between the first day of the first chemotherapy course till progression or relapse (for TTP), or till last known to be alive or death (for OS). The cut-off date for follow-up was June 19th, 2019.

Exploratory subgroup analyses were performed based on disease type (intrahepatic vs other), disease extent (locally advanced vs metastatic) and obtainment of secondary resection.

Safety endpoints included toxicity occurrence and severity (NCIC-CTCAE), as well as treatment duration and delivered dose intensities of the three cytotoxics. All analyses were performed with intent-to-treat using SPSS® v18.0 software (Chicago, IL, USA).

## Results

### Patients’ characteristics

**Fig. 1:**
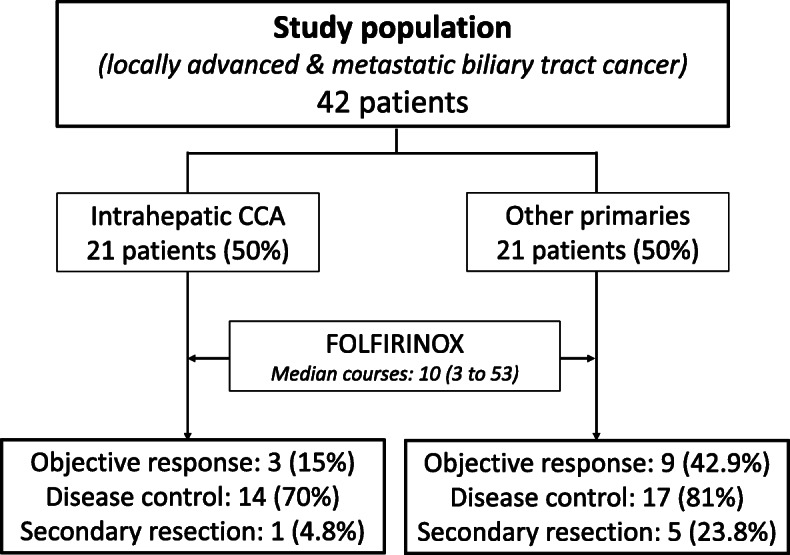
Study Flowchart

Between December 2013 and November 2017, a total of 42 consecutive patients received at least one cycle of FOLFIRINOX for advanced or metastatic BTC in our center and were included in the current analysis (Fig. 1). Patients’ main characteristics at baseline are reported in Table 1. All of the patients had a good general condition (WHO Performance Score of 0 or 1) and were younger than 85 years of age. Their cancer was locally advanced in 10 patients (23.8%) and metastatic in 32 patients (76.2%). All of the 14 patients with obstructive jaundice at initial assessment underwent endobiliary stent placement before treatment onset.

**Table 1:** Main characteristics of all 42 patients with advanced biliary tract cancer

Characteristics	Number of pts (***N*** = 42)
**Sex**	
Male	17 (40.5%)
Female	25 (59.5%)
**Age (years)**	
Median (range)	67 (36–84)
≤ 65	18 (42.9%)
> 65	24 (57.1%)
**WHO PS**	
0	23 (54.8%)
1	19 (45.2%)
**Primary tumor location**	
Intrahepatic CCA	21 (50.0%)
Other locations:	21 (50.0%)
Gallbladder	8 (19.0%)
Perihilar CCA	7 (16.7%)
Distal CCA	4 (9.5%)
Ampulloma	2 (4.8%)
**Disease extension**	
Locally advanced (LA)	10 (23.8%)
Metastatic (M)	32 (76.2%)
**Site of metastases**	
None (LA)	10 (23.8%)
M in liver only	11 (26.2%)
M in liver + other sites	15 (35.7%)
M in other sites only	6 (14.3%)
**Biliary stent placed**	14 (33.3%)

### Treatment features

FOLFIRINOX regimen started with all of the three cytotoxic agents in 31 patients (74%). Irinotecan was introduced in the protocol after 1 to 3 initial courses without it because of high level of serum bilirubin (> 1.5x ULN) in 11 patients (26%).

**Fig. 2:**
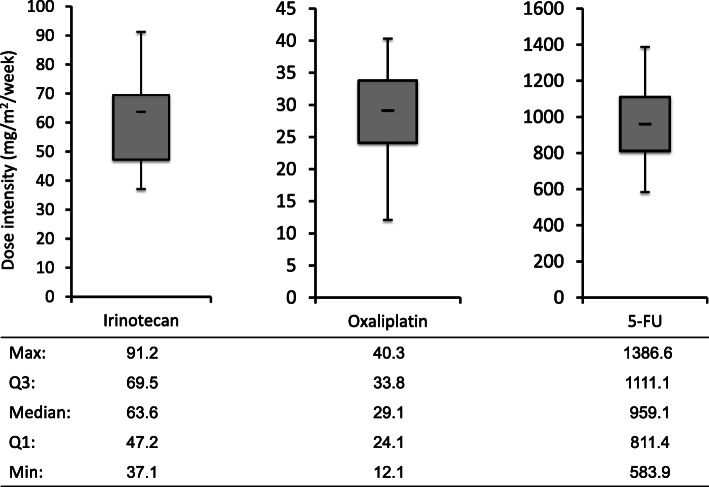
Actual dose-intensities of 5-Fluorouracil, Oxaliplatin and Irinotecan administered

A total of 542 courses of FOLFIRINOX were given, resulting in a median number of 10 courses per patient (range: 3 to 53) with median treatment duration of 6.3 months (1.4 to 26.8 months). The median actual dose-intensities (mg/m^2^/week) over the six first courses were 63.6 (range, 37.1–91.2) for irinotecan, 29.1 (range, 12.1–40.3) for oxaliplatin and 959.1 (range, 583.9–1386.6) for 5-Fluorouracil (Fig. 2). Thus, the median relative dose-intensities were 70.7% (range, 41.2–101.4) for irinotecan, 68.4% (range, 28.4–94.9) for oxaliplatin, and 68.5% (range, 41.7–99.0) for 5-Fluorouracil.

### Treatment safety

Treatment with patient-tailored FOLFIRINOX was well tolerated, with no occurrence of toxic deaths or emergency admissions for treatment toxicity. Thus, no patient reported any grade 3 or more clinical toxicity, except for cumulative peripheral sensory neuropathy, which then led to oxaliplatin discontinuation in 13 patients (31%). Hematological and biochemical toxicities were also chiefly moderate and uncomplicated, with no occurrence of febrile neutropenia. Thus, the most frequent Grade 1–2 toxicities encountered included fatigue, diarrhea, anorexia, nausea/vomiting, mucositis, alopecia, abdominal pain, hand-foot syndrome and allergic reaction. Chemotherapy was withheld for as long as clinically indicated during the treatment of disease-related biliary complications, all of which were not serious.

### Efficacy outcomes

**Fig. 3:**
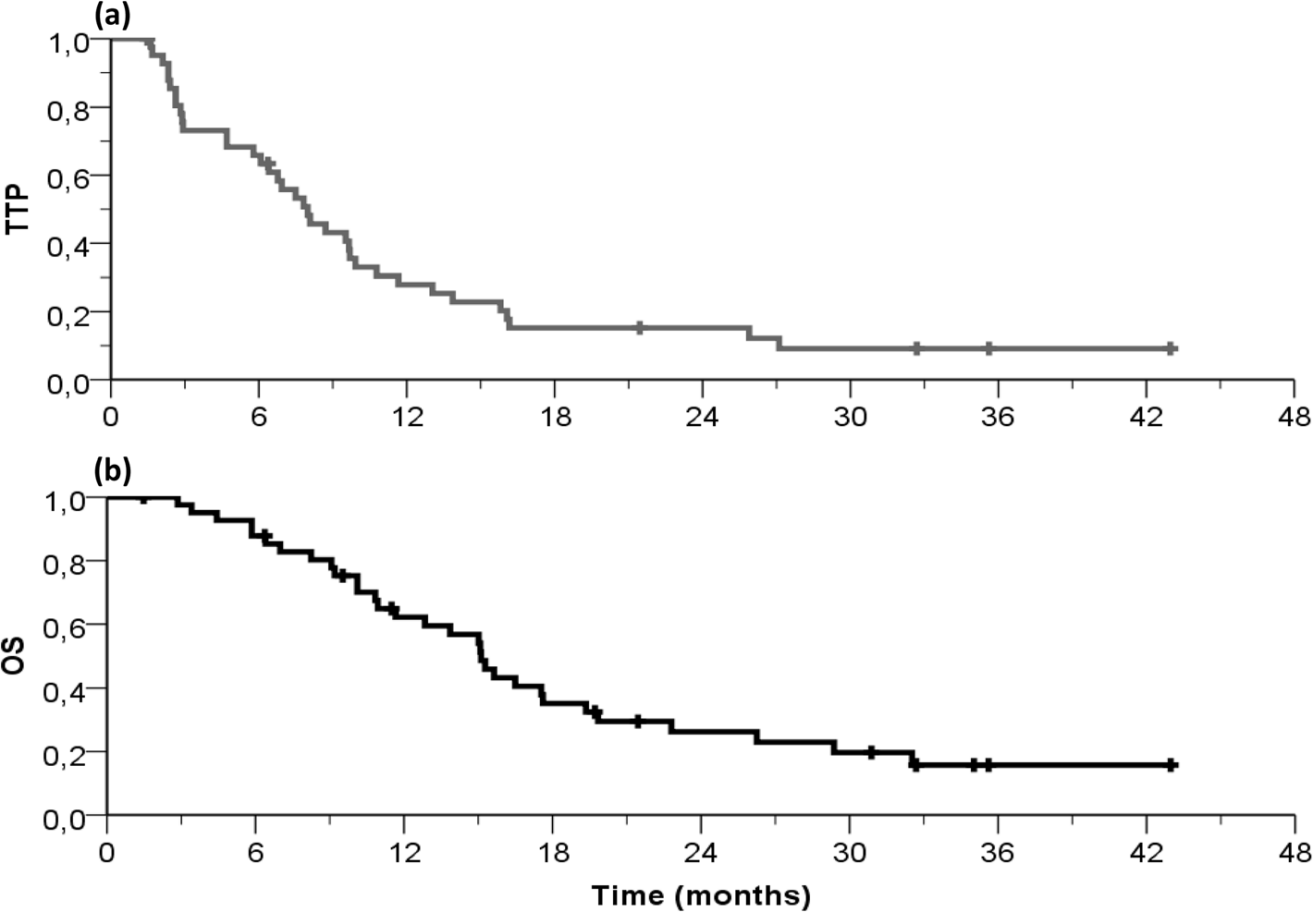
Kaplan-Meier curves showing time to progression (**a**) and overall survival (**b**) in the whole study population

In the 41 evaluable patients, 12 obtained an objective response and additional 19 had their disease stabilized. This resulted in objective response (ORR) and disease control (DCR) rates of 29% [95%CL, 16–43] and 76% [62–90] respectively. Six out-of 12 responders (50%) underwent secondary surgery with macroscopic complete resection. After a median follow-up of 14 months (range, 2 to 43), median intent to treat TTP and OS were respectively 8.0 months [5.8–10.1] (Fig. 3a) and 15.1 months [13.0–17.2] (Fig. 3b), and no cancer-unrelated death had occurred.

### Subgroup analyses

**Fig. 4:**
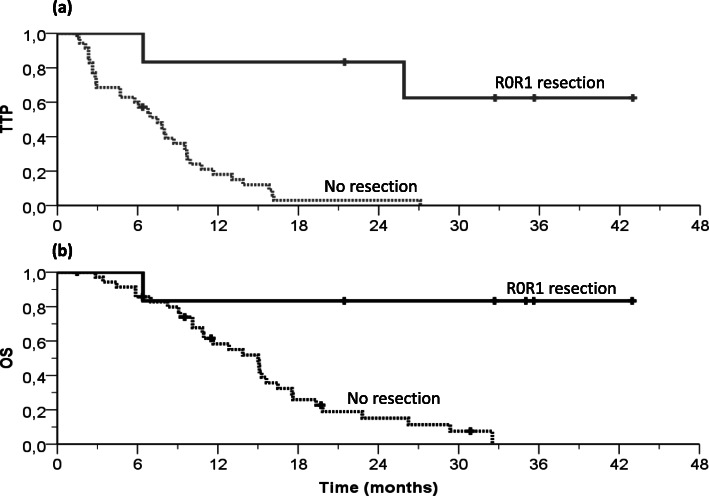
Time to progression (**a**) and overall survival (**b**) curves in the subgroup of 6 patients who underwent secondary surgical resection of downsized disease

Patients with IHCC accounted for half of our study population (*N* = 21). The ORR, DCR and secondary surgical resection after downsizing in this subgroup were 15.0, 70.0 and 4.8%, respectively. Corresponding figures in the remaining 21 patients with other biliary tract tumors (Table 2) were 42.9% for ORR, 81.0% for DCR, and 23.8% for secondary resection. Median TTP according to primary tumor site was 6.9 months in IHCC and 11.7 months in the others. Observed median OS duration was 15.3 months in intrahepatic tumors and 15.1 months in the remaining subgroups. When considering disease extent, ORR, DCR, secondary surgery, median TTP and OS were 30.0, 80.0, 30.0%, 4.7 and 9.1 months, respectively, in the 10 patients with locally advanced disease. Corresponding figures in the 32 patients with stage IV disease were 29.0, 74.2, 9.4%, 9.5 and 15.1 months, respectively. Somewhat expectedly, the 6 patients for whom secondary surgical resection of residual disease after downsizing with FOLFIRINOX was deemed indicated at the onco-surgical multidisciplinary team meeting displayed excellent outcomes, as shown in Fig. 4a for TTP and in Fig. 4b for OS.

**Table 2:** Efficacy according to primary tumor location and disease extension

		ORR	DCR	R0R1 resection	TTP, median, months [96%CL]	OS, median, months [96%CL]
**Overall study population** *N = 42*^a^		12 (29.3%)	31 (75.6%)	6 (14.3%)	8.0 [5.8–10.1]	15.1 [13.0–17.2]
Primary tumor location	Intrahepatic CCA *n = 21*^a^	3 (15.0%)	14 (70.0%)	1 (4.8%)	6.9 [4.1–9.8]	15.3 [8.8–21.7]
	Other locations *n = 21*	9 (42.9%)	17 (81.0%)	5 (23.8%)	11.7 [4.1–19.2]	15.1 [13.0–17.2]
	*p*-value	**0.050**	0.484	0.184	**0.005**	0.492
Disease extension	Locally advanced *n = 10*	3 (30.0%)	8 (80.0%)	3 (30.0%)	4.7 [1.1–8.3]	9.1 [0–19.2]
	Metastatic *n = 32*^a^	9 (29.0%)	23 (74.2%)	3 (9.4%)	9.5 [7.1–11.9]	15.1 [13.2–17.0]
	*p*-value	1	1	0.135	0.479	0.932

^*a*^*1 patient was lost to follow up before the first evaluation. Thus, for computing ORR and DCR, it was considered 41, 20 and 31 patients for overall, intrahepatic CCA and metastatic group respectively*

## Discussion

The results of our retrospective study show that patient-tailored FOLFIRINOX results in promising efficacy and no new safety issues among patients with advanced BTC. With mature follow-up data, we observed a median TTP of 8.0 months and a median OS of 15.2 months in the overall intent-to-treat population. These outcomes appear favorable compared with those series of metastatic BTC treated with the current gold-standard regimen combining gemcitabine and cisplatin, resulting in median PFS and OS of 8.0 and 11.7 months, respectively [8, 24–28]. Furthermore, the strategy of intensifying the association of gemcitabine and a platinum compound with an anti-EGFR monoclonal antibody did not result in enhanced antitumor activity [25]. Conversely, nano-albumin-bound paclitaxel added to gemcitabine and cisplatin seemed to provide encouraging efficacy, albeit with more frequent severe toxicity [29]. Instead, the patient-tailored FOLFIRINOX regimen used here confirmed its satisfactory tolerance we previously reported in advanced pancreatic adenocarcinoma [13], with no new safety issues observed in comparison with existing literature [17, 18, 30]. We speculate that the patient-tailored approach we adopted here, which recapitulates similar ones with FOLFIRINOX in pancreatic adenocarcinoma [13, 31, 32] displayed favorable tolerance profile thanks to the deal made between the decreased dose intensity of the three cytotoxics and the global duration of treatment, somewhat allegedly avoiding over- or under-exposure to the drugs [33]. Thus, median actual delivered dose intensities were of the order of ~ 70% (Fig. 2), a figure overall comparable to literature data on the same combination in pancreatic adenocarcinoma [34, 35].

Additionally, the adequate antitumor activity resulted from upfront patient-tailored FOLFIRINOX in our study (overall ORR of 29%, raising to 43% in extra-hepatic localisations), alongside an enduring onco-surgical multidisciplinary approach refined in liver metastases from colorectal cancer [36, 37], permitted to achieve secondary resection after down-sizing of initially unresectable disease in about half of the responders. The proportion of patients whose disease shrank noticeably following FOLFIRINOX administration and underwent secondary resection was higher in the subgroup with EHCC (Table 2). Whichever the primary localisation, the outcomes of these patients withstanding the onco-surgical approach (Fig. 4) was considerably better than most of the real-world experience reports in advanced BTC [1, 38, 39]. Furthermore, recent evidence suggests that the combination of liver-targeted locoregional interventions with systemic treatment in patients with IHCC could provide better chances of secondary resection (~ 11–22%), hence of better outcomes [27, 28]. Indeed, our experience in patients with unresectable intrahepatic localisation, which is notoriously associated with poorer prognosis [26], compares favorably with reports from other tertiary centres [40]. Moreover, the triplet combination of irinotecan, oxaliplatin and 5FU can be effectively and safely administered via the hepatic artery to maximize its therapeutic index [41]. Last but not least, replacing 5FU by an oral fluoropyrimidine such as S-1 can render this regimen more convenient in selected patients with comparable efficacy outcomes [42].

Notwithstanding, we acknowledge the limitations in generalizability of our findings, given the possible selection bias related to the fittest patients deemed likely to tolerate the aggressive regimen of FOLFIRINOX and to the single-institution experience in an academic tertiary centre for both medical oncology and hepato-biliary surgery. Thus, this retrospective report on real-life practice should warrant prospective studies to tackle these limitations like the ongoing French phase II/III randomized trial comparing modified FOLFIRINOX versus CisGem [15].

## Conclusions

Despite the overall poor prognosis of patients with unresectable cholangiocarcinoma confirmed here, real-world experience with patient-tailored frontline FOLFIRINOX resulted in promising outcomes for both efficacy and safety. Additionally, this active regimen could constitute the groundwork for establishing optimal onco-surgical approaches, hepatic-targeted locoregional interventions, and possibly sequential systemic chemotherapeutic avenues.

## Data Availability

The datasets used and/or analysed during the current study are available from the corresponding author on reasonable request.

## Abbreviations

BTC: Biliary tract cancer
LA: Locally advanced
M: Metastatic
OS: Overall survival
TTP: Time to progression
ORR: Overall response rate
DCR: Disease control rate
CCA: Cholangiocarcinoma
IHCC: Intrahepatic cholangiocarcinoma
